# Intra-Abdominal Localisation of a Buschke-Lowenstein Tumour: Case Presentation and Review of the Literature

**DOI:** 10.1155/2013/187682

**Published:** 2013-09-18

**Authors:** N. E. Wester, E. M. Hutten, C. Krikke, Robert A. Pol

**Affiliations:** Division of Transplantation Surgery, Department of Surgery, University Medical Center Groningen, University of Groningen, P.O. Box 30 001, 9700 RB Groningen, The Netherlands

## Abstract

Giant condyloma acuminatum or Buschke-Lowenstein tumour is a very rare disease which usually is located in the genital, anorectal, and perianal regions. It is regarded as a type of verrucous carcinoma occurring on anogenital mucosal surfaces where it is locally invasive but displays a benign cytology. We describe a case of a 24-year-old woman with persisting condyloma acuminata progressing to a large intra-abdominal Buschke-Lowenstein tumour. To our knowledge such an advanced stage has only been reported once before. The severity and extent of the tumour both determine the treatment and patient outcome. Treatment was impeded by cachexia, an immunosuppressive state after kidney transplantation and difficulties in establishing a reliable diagnose. Interferon treatment was started which initially led to tumour reduction but was complicated by an interferon-induced pancreatitis, pneumonia, and fasciitis necroticans resulting in death. We present a literature overview on the treatment options for a Buschke-Lowenstein tumour, with emphasis on interferon therapy, with all the advantages and disadvantages.

## 1. Introduction

Worldwide prevalence of human papilloma virus (HPV) is 10%, mostly affecting women under the age of 35 [1]. The associated condylomata acuminata (CA) has an estimated prevalence of 0.75–3% in Europe and 1% in the United States [2]. One of the risk factors for CA is an immune-compromised state. On rare occasions progression to a Buschke-Lowenstein tumour (BLT) occurs. A BLT is a very rare, slowly growing, locally destructive, and infiltrative tumour associated with HPV types 6, 11, 16, and 18. Usually the tumour is located in the genital, anorectal, and perianal regions. Despite the benign histological characteristics of the tumour there is a high risk of recurrence and malignant transformation to squamous cell carcinoma (SCC), in particular for HPV types 16 and 18 [3–7]. Little is known about the true incidence rates of BLT due to the difficulties in histomorphological differentiation and thereby making the correct diagnosis. Current literature reports an increase to 6.3 cases per year in the last decade [8]. Due to this low incidence little is known about treatment outcomes, making comparison between various treatment regimens difficult.

To date only three papers are available reporting on, respectively, 42, 63, and 51 cases [3, 8, 9] and one small review [10]. They conclude that complete resection is the preferred initial therapy as it has the highest success rate and the lowest risk of recurrence. Contradictory is that surgery can be very mutilating as extensive resection is usually required due to ingrowth into local structures. Alternatively chemotherapy and radiation therapy have been described with varying results. However, in most cases, it proved to be difficult to determine a true malignancy so that there is a risk of overtreatment. 

A possible alternative treatment may be interferon therapy as an effort to boost the patient's own immune system [1–16]. As a new and experimental treatment for targeting BLT, there are no large series available let alone randomized trials. The majority of available data comes from other case reports and small case series concerning CA [17–20]. We present a rare case and provide a literature overview on the treatment options for a BLT, with emphasis on interferon therapy.

## 2. Case Presentation

**Figure 1:**
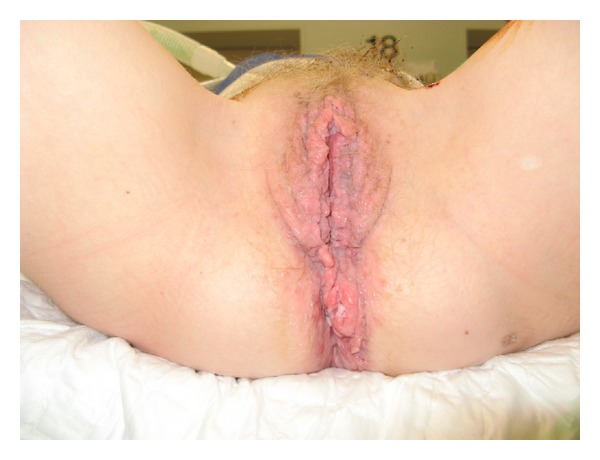
Extensive condyloma acuminata of the vulvar and perianal regions.

**Figure 2:**
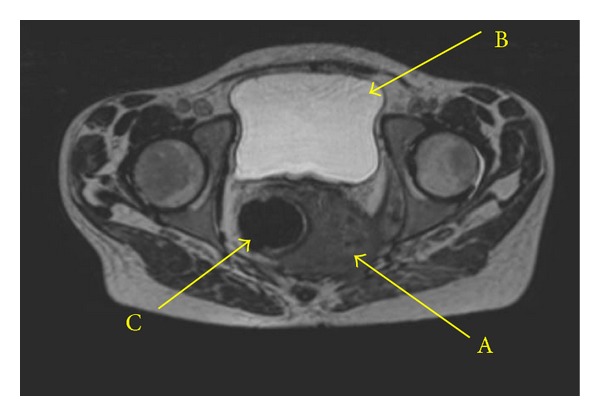
MRI scan of the small pelvic. A mass, originating from the perianal region, is expanding along the endopelvic fascia to dorsolateral with ingrowth in the left m. obturatoria and m. piriformis and ventral in the m. gluteus (Capitol A). No bladder ingrowth visible (Capitol B). The mass does have a direct relation with the wall of the lower rectum (Capitol C).

A 24-year-old cachectic female patient, with a status after kidney transplantation due to hemolytic-uremic syndrome in 2000, was referred to our university medical center because of persisting incapacitating CA under prolonged immunosuppressive therapy. Her initial immunosuppression regimen consisted of tacrolimus 5 mg and prednisolone 7.5 mg daily. At presentation in 2010 she suffered from severe CA located both intravaginal and perianal (Figure 1). This has led to excessive pain and defecation problems despite local and topical treatment by surgeons, gynaecologists, and dermatologists for over five years. Viral serology revealed the patient to be HPV-16 and HPV-18 positive. Her HIV status was negative. At our center multiple biopsies revealed serious dysplasia, and only one sample revealed malignant cells. Because of persistent lower back pain a MRI scan was performed. This showed a large process in the lower pelvic region with extension into the abdomen and cavum douglasi, consistent with the diagnosis intra-abdominally located BLT (Figure 2). Prior to presentation at our hospital, the patient already had, in vain, several surgical procedures to obtain local control. At presentation both the cachexia of the patient, the localisation and infiltration of the process made surgical treatment (anterior and posterior exenteration) no realistic option. As a malignancy could not be definitely established, radio- or chemotherapy was therefore not indicative. Because the tumour had centrally liquefied, resulting into abscesses, multiple drainage procedures were necessary. In order to treat the BLT the immunosuppressive therapy was lowered to 5 mg prednisone daily, and interferon therapy was started as an effort to boost the patients' immune system [12, 15, 16]. After multidisciplinary consultation a dosage of 180 *μ*g/week interferon alfa 2B was given by subcutaneous injections. After it became clear that the kidney function remained stable, the immunosuppressive therapy was further lowered to 5 and 2.5 mg prednisolone every other day. Because of severe malnutrition and cachexia, the patient received gastric tube feeding. During the interferon therapy a new abscess arose in the lower back, which was found to have a direct relationship with the BLT. On a follow-up MRI and CT scan, respectively, two and four months after the first interferon injection, a reduction in tumour mass was visible. As a chance finding an interferon-induced pancreatitis was visible on the CT. There were no further adverse effects of the therapy. Based on the established tumour reduction, interferon therapy was continued. Unfortunately after four months of treatment the patient was readmitted to our hospital because of a pneumococcal pneumonia for which antibiotic therapy was started. During hospitalization the patient developed progressive pain in the lower back and left leg in combination with a septic profile. The diagnosis necrotizing fasciitis was made and due to the clinical condition of the patient, combined with the extent of the disease, the treatment was discontinued in agreement with the family and the patient died. Unfortunately no informed consent was obtained for a postmortem examination.

## 3. Discussion

This report underlines the impact and difficulties of treating BLT. Progression to an intra-abdominal mass even further complicates treatment and to date has been described only once. Early detection of abdominal involvement in combination with a reliable histopathological diagnosis is essential for determining the appropriate treatment and predicting treatment outcome. When confronted with possible BLT it is very important to exclude an underlying malignancy. Other pelvic malignancies (gynaecological, gastrointestinal, and urological) should be considered, especially in younger women and patients with HPV types 16 and/or 18. In transplantation patients, posttransplant lymphoproliferative disorder should also be excluded, which was done in this patient and turned negative. In large BLTs possible sampling errors can make reliable detection of a malignancy difficult as they can contain malignant, premalignant, and/or benign cells. Also in our case only one biopsy sample revealed malignant cells. Repetitive biopsies were never able to definitely demonstrate true malignant transformation.

This report also shows that the choice of treatment is highly dependent on patient factors, such as cachexia and immune state in our patient. In this case abdominal involvement became apparent in an already advanced stage. With this paper we hope to create more awareness for the complexity of this disease and the considerations that must be taken. 

A BLT is a rare condition, and, although the incidence appears to be increasing, there remains a lack of evidence on the appropriate steps to take in the treatment of these patients [8]. The tumour has a considerable risk of malignant transformation into fully invasive SCC or verrucous carcinomas [1, 7]. The disease is thereby associated with high recurrence and mortality rates of, respectively, 67 and 21 percent [3]. One of the main risk factors for the development of a BLT is an immune-compromised state. The cell-mediated immune response is essential in the natural control of HPV infections [12, 22]. Immunosuppressive drugs increase the risk of posttransplant cancer. Inparticular cyclosporines reduce UV-induced DNA repair by peripheral blood mononuclear cells [23, 24]. Without prompt and adequate treatment of a BLT there is a risk of local invasiveness leading to various complications such as abscess formation, fistulas, and defecation problems by ingrowth in or pressure to the rectum [3, 9]. In current literature only one case has been described with a similar intra-abdominal mass as in our patient. In this particular case extend of the disease was only detected during surgery [25]. In order to avoid this it has been suggested to perform a CT scan prior to surgery to be properly informed on the extent of the disease [26]. In this case the scan proved to be of great value. 

When treatment is considered, the first choice is surgery in which a radical resection should be achieved [3, 5, 8, 9]. Despite radical or wide local excision the tumour still has a high recurrence rate of 50% [3]. The possibilities for surgery, however, are sometimes limited by factors as a poor baseline condition and advanced disease due to patient or doctors delay [10–13, 16]. Surgical therapy often requires extensive resections by means of anterior and posterior exenteration or even a pelvectomy [12, 27].

Chemo- and/or radiotherapy have been applied in many different ways, usually following unsuccessful surgical treatments. If an underlying malignancy can be established, radio- and/or chemotherapy as main treatment or prior to surgery [27–32] seem to have good results [9]. However, when used after multiple surgical interventions or as adjuvant therapy, results are less favorable [3, 33]. The detection or exclusion of malignant transformation is essential before initiating therapy but can be very difficult to obtain. In an early stage of the disease sampling error can easily occur. Representative biopsies herein can be decisive in the establishment of a malignancy [12, 34]. Only one report showed good results with chemoradiation as adjuvant therapy prior to surgery without first verifying a malignancy [25]. This is however a rather controversial approach and can therefore not be recommended as initial treatment.

Another controversial alternative to surgery or radio- chemotherapy is the administration of interferon. Interferon has been described as a treatment for CA and BLT when other treatments have been excluded or unsuccessful. The published results with this treatment are very contradictory and vary from a complete lack of effect [10, 11, 13, 14, 17] to a reduction of the disease [12, 15, 16, 19, 35]. A recent meta-analysis unfortunately showed no superior effect of imiquimod (interferon *α*) compared to podophyllotoxin in the treatment of CA [20]. Only few side effects of interferon are described in literature varying from miscellaneous reactions as fever, chills, headache, myalgia's, and leucocytosis to more severe and life threatening side effects as pericarditis, autoimmune disease and myelosuppression [16, 17, 19, 36–38]. When considering treatment with interferon there is a lack of consensus in dosage, frequency, and length of treatment (Table 1) [10–16, 21]. We encountered the same problem and after extensive multidisciplinary consultation decided to a dosage of 180 *μ*g/week interferon alfa 2B by subcutaneous injections. As tumour reduction had been established during follow-up CT, an appropriate dose-effect relationship was assumed. Due to the short follow-up this cannot be determined with any certainty. 

This case has a few shortcomings. As mentioned in the introduction, surgery is considered first-choice treatment. Unfortunately at the time of presentation at our hospital the BLT had already grown into adjacent structures. Whether extensive resection or debulking still would have had a positive outcome cannot be assessed. Another consideration could have been to suspend all immune suppressant therapy instead of just lowering the dosage. Ceasing the prednisolone could possibly have led to a better effect of the interferon therapy and prevented the fasciitis. Furthermore, immunosuppressant drugs such as sirolimus were not considered. This could have had a positive contribution because it has antiproliferative properties. Also, considering the high costs of interferon therapy, the lack of consensus on the appropriate form of administration and dosage, and the potentially serious side effect, this certainly cannot be recommended as standard therapy.

In conclusion, we report one of the first cases of a large intra-abdominally located BLT. We have tried to provide a practical overview of current treatment options, which may benefit future patients. The outcome of each treatment depends on the disease stage, differentiation, and patient characteristics. Although in this case surgery was no longer a realistic option it should be considered the gold standard. Interferon therapy is a costly and still experimental treatment and should be regarded as a last resort. Patients should be very thoroughly informed about the potential life-threatening risks.

## Figures and Tables

**Table 1:** Literature overview of interferon therapy.

Article	Patient characteristics		Tumour characteristics			Interferon (IFN) therapy								Outcome
Author (year of publication)	Gender, age	Comorbidity	Tumours ze (cm)	Location	HPV type	Initial therapy (earlier surgical interventions Yes/No)	Followed by	Type	Way of administration	Dose	Frequency	Length	Adverse effects	
Tan et al. (2010) [15]	F, 21	immuno-therapy (SLE)	15 × 7 × 3	Vulvar	6	Radical resection and IFN (Y)	none	*α*-2	—	—	—	6 months	—	CR (2 years of follow-up)
Mudrikova et al. (2008) [10]	M, 44	HIV	—	Perineal and perianal	6	IFN + local cidofovir* (Y)	none	Pegylated	Subcutane	—	—	—	None	Pt died
De Toma et al. (2006) [21]	M, 46	None	8 × 6 × 6	Perianal	6	Surgical local excision** (N)	IFN	—	Systemic	3 MU	—	—	—	CR (3 years of follow-up)
Antony et al. (2003) [11]	M, 61	Therapy-resistant erythroderma	—	Perineum involving the penis	16	IFN* (N)	Chemo-therapy	*α*	Subcutane	9 MU	3/wk	—	Neutropenia - could be chemoinduced	No effect, patient died of septicaemia
Geusau et al. (2000) [12]	M, 40	None	—	Perianal	6	IFN (Y)	Partial chirurgic resection	*α*-2b	Intralesional	10 MU	3/wk	28 months	—	CR after 12 m
Grassegger et al. (1994) [13]	M, 42	None	—	Genital/ perianal	6	Surgery (Y)	IFN	*α*-2c	Subcutane	2 MU	6/wk every 2nd wk	3 months	—	Recurrence after 2 y
Tsambaos et al. (1994) [16]	M, 39	None	8,7 × 7,3 × 5,6	Inguinal	6, 11	IFN* (N)	Podofyline	*α*-2b	Intralesional	9 MU	3/wk	—	Fever, myalgias, chills	CR after 5 months (16 months follow-up)
Gritsch et al. (1989) [14]	M, 29	None	3,5 × 4	Perianal	—	IFN (Y)	Partial chirurgic resection	*α*	Subcutane	6 MU	7/wk	30 days	—	Progression, patient died of pneumonia

(—): If not mentioned in article. *Patient refused surgery, **Followed by radiotherapy and surgery, CR: Complete remission.
